# Development of a Handheld Line Information Reader and Generator for Efficient Management of Optical Communication Lines

**DOI:** 10.3390/s17091950

**Published:** 2017-08-24

**Authors:** Jaeyul Lee, Hyungwoo Kwon, Jaewon Song, Mansik Jeon, Jeehyun Kim

**Affiliations:** 1 School of Electronics Engineering, College of IT Engineering, Kyungpook National University, 80 Daehak-ro, Buk-gu, Daegu 41566, Korea; jaeyul@knu.ac.kr (J.L.); jwsong@knu.ac.kr (J.S.); jeehk@knu.ac.kr (J.K.); 2 TDI Co. Ltd., 80 Daehak-ro, Buk-gu, Daegu 41566, Korea; hwkwon@tdi.co.kr

**Keywords:** line information reader, line information generator, optical communication, fiber networks, optical line management

## Abstract

A handheld line information reader and a line information generator were developed for the efficient management of optical communication lines. The line information reader consists of a photo diode, trans-impedance amplifier, voltage amplifier, microcontroller unit, display panel, and communication modules. The line information generator consists of a laser diode, laser driving circuits, microcontroller unit, and communication modules. The line information reader can detect the optical radiation field of the test line by bending the optical fiber. To enhance the sensitivity of the line information reader, an additional lens was used with a focal length of 4.51 mm. Moreover, the simulation results obtained through BeamPROP^®^ software from Synopsys, Inc. demonstrated a stronger optical radiation field of the fiber due to a longer transmission wavelength and larger bending angle of the fiber. Therefore, the developed devices can be considered as useful tools for the efficient management of optical communication lines.

## 1. Introduction

The market for optical networks has grown by diversifying the routes of optical fiber communication. The density of fiber lines has grown along with an exponential increase in data traffic and data transmission [1,2,3]. The capacity of commercial light-wave systems increased from roughly 1 Gb/s in the mid-1980s to 1 Tb/s by 2000 [4]. Growth of the traffic and data transmission will result in a requirement for interfaces to the networks [5,6]. Many countries have adopted fiber-to-the-home (FTTH) and have started to use optical fiber networks such as active optical networks (AONs) or passive optical networks (PONs) [7,8,9,10]. The FTTH application has increased because of the growth of Internet subscribers [11]. The application of wavelength division multiplexing (WDM) has affected the growth of optical networks [12,13,14]. WDM can use multiplexing technology in optical communication networks [15], and monitoring of linear and non-linear signals distortion are studied for more efficient and reliable optical network performances [16,17,18].

In this situation, many researchers have diversely studied optical network management and monitoring systems. Loss and power, network state, signal-to-noise ratio (SNR), and Q-factor are managed and monitored with the development of optical fiber sensors and optical time domain reflectometry (OTDR) techniques [19,20]. Optical performance monitoring (OPM) is an inevitable process for managing optical fiber conditions and finding the positions of faulty fibers. OPM is anticipated to enhance the capacity of optical networks as well as their survivability and re-configurability [21,22,23]. Network faults can cause the disruption of communication services and severe data loss [24]. Thus, fault line detection and the management of line information are considered essential requirements. Survivability techniques and monitoring cycles with heuristic depth-first searching and shortest-path Eulerian matching are used for fault detection and path performance monitoring [24,25]. In addition, many mechanisms and algorithms have been utilized in a variety of methods for optical line terminals, optical network units, and tunable OTDR [26,27,28]. However, the network lines are not properly managed in online and offline modes. Normally, hundreds of network fiber lines are passed through an optical fiber distribution (OFD) in a square tray. A twist phenomenon of the lines easily occurs between OFDs. Line twisting causes work confusion during fault management, which can result in increased work time for repair efforts and difficulty in maintaining management system transparency. For this reason, communication network industries are suffering from managing and monitoring network lines in optical communication networks [22]. Conventionally, sticky notes, barcodes, and scratch paper have been used to classify and manage network lines. The conventional examination method increases artificial errors in communications by pulling or intensely bending each optical fiber [21]. Most fault line management is conducted in the middle of the night, when there is less network traffic, because of the artificial errors in communications. This often introduces human error by the misconnection of the lines and causes the problems of increased labor costs and work times.

In this study, we developed a handheld line information reader and line information generator to detect faulty lines and manage optical networks in online and offline modes. When a fault occurs in an optical line, the position can be found by using OTDR and geographic information systems (GISs) [29,30,31]. The line information generator modulated a transmission signal by using on–off key optical method. The on–off key optical system is not popular in current optical networks. Nevertheless, we adapted the on–off key modulation to the proposed setup by considering the simple handling of the optical lines, although quadrature phase shift keying (QPSK), quadrature amplitude modulation (QAM), phase shift keying (PSK), etc. are more utilized in recent communication systems for higher-order modulation format [32,33,34]. The line information generator is capable of generating a signal for each line, and the line information reader detects that signal from the optical radiation field by bends in the fiber. The simulation results are presented using the developed device principle. Simulation of an optical radiation field was executed by radius of curvature and wavelength of transmission [35,36,37]. The sensitivity of the handheld line information reader was enhanced by employing various focusing lenses. Therefore, the developed instrumentation devices and methods can be an effective and cost-efficient tool in optical network line management and monitoring systems.

## 2. Experimental Setup and Methods

**Figure 1:**
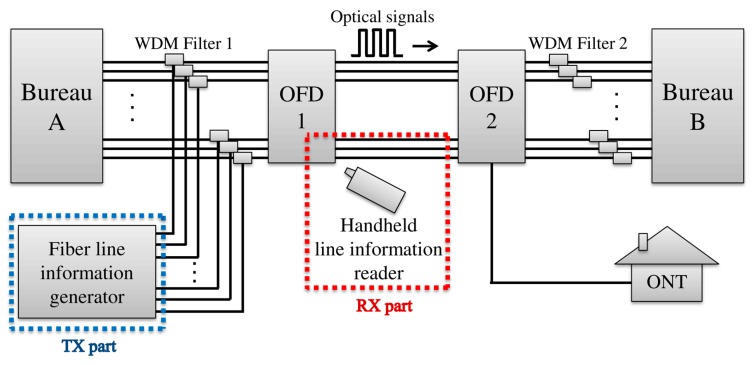
Schematic of the developed fiber line monitoring and management system in optical communications. OFD: optical fiber distribution, ONT: optical network termination, WDM: wavelength division multiplexing, TX: transmitter, RX: receiver.

A schematic of the developed fiber line monitoring and management system is illustrated in Figure 1. In the developed system, first, the fiber line information generator is installed to transmit the modulated signals along with a bureau. Because communication data are transmitted between Bureau A and Bureau B through the fiber lines, error lines in the OFD should be identified. The fiber line information generator transmits modulated signals of 1625 nm wavelength to distinguish each line. The modulated signals are combined with the communicated data of Bureau A by WDM Filter 1. The protocol of the modulated signals is matched with the protocol of the line information reader. To avoid cross-communication with the network communication wavelength, the signal is modulated with an out-band wavelength of 1625 nm, which is greater than the network communication wavelength of 1270 nm. The handheld line information reader is used to detect the error lines in the OFD when the combined signals transmit towards Bureau B. When the radiation field radiates by bends in the fiber lines, the modulated signals of the radiation field are detected by the line information reader. The modulated signal is demodulated to the line information by the matched protocol with the line information generator. In addition, changes in signal intensity provide a reference of judgment for the line condition. As a result, the line information can be monitored and managed by the developed system.

**Figure 2:**
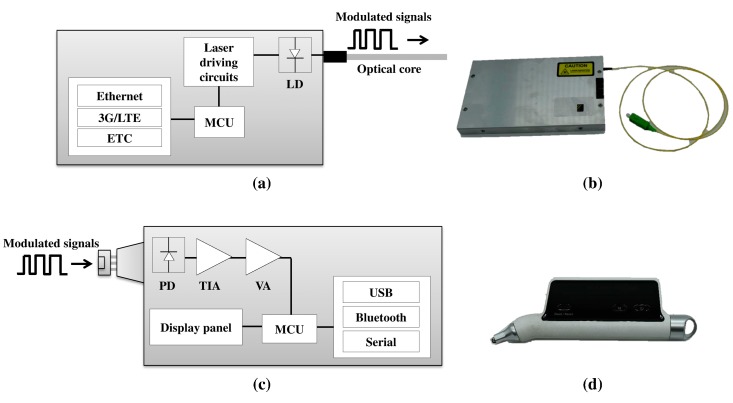
(**a**) Schematic of the TX part, (**b**) photograph of the TX part, (**c**) schematic of the RX part, and (**d**) photograph of the RX part. LD: laser diode, MCU: microcontroller unit, 3G: third-generation, LTE: long-term evolution, ETC: et cetera, PD: photo diode, TIA: trans-impedance amplifier, VA: voltage amplifier.

Detailed descriptions of the transmitter (TX) and receiver (RX) parts are shown in Figure 2. A schematic of the fiber line information generator (i.e., the TX part for transmitting the line information) is shown in Figure 2a, and a photograph of the developed device is shown in Figure 2b. The TX part consists of a microcontroller unit (Atmel Corporation ATmega16(L), San Jose, CA, USA); laser driving circuit (Infineon Technologies AG IRLML2502, El Segundo, CA, USA); laser diode, which distributes feedback with a 1625-nm laser; and third-generation/long term evolution (3G/LTE) modules. In the TX part, the microcontroller unit controls the laser driving circuit and generates the modulated digital signals from 1625 nm to 1670 nm by adapting the internal modulation method. The characteristics of the transmitted signals is digitally on–off key modulation. The 50 μs time period of the transmitted signal means zero, and the 100 μs time period of the transmitted signals means one. The transmission signals can be digitally modulated by switching the pattern of the signal period, and the receiver detects the modulated signals, which are modulated by measuring the time of high and low power through the photodiode. Thus, the characteristics of the transmission signals impact the performance of the receiver. The communication module receives the order of the RX part. A schematic of the fiber line information reader (i.e., the RX part for receiving the line information) is shown in Figure 2c. A photograph of the developed device is shown in Figure 2d. The RX part consists of a photo diode, trans-impedance amplifier (Texas Instrument Incorporated OPA209, Dallas, TX, USA), voltage amplifier, microcontroller unit (Atmel Corporation ATmega16(L), San Jose, CA, USA), display panel (Newhaven Display International, Inc. NHD-0220CW-AY3, Elgin, ND, USA), and USB (Future Technology Devices International Ltd. FT230XS-R, Glasgow, UK) and Bluetooth (Arduino AG HC-06, Turin, Italy) modules. The RX part has a wavelength detection range from 1200 nm to 1700 nm. Normally, conventional transmission bandwidths were S-band (1460 nm to 1530 nm), C-band (1530 nm to 1565 nm), and L-band (1565 nm to 1625 nm) in optical communication networks. However, the RX part can detect the conventional bandwidth and additional bandwidth. It can be applied the other transmission bandwidths in the future. In the RX part, the modulated signals are detected through the photo diode and the current is converted to voltage by the trans-impedance amplifier. The voltage is amplified by the voltage amplifier. Then, the microcontroller unit identifies the line information by the interval and intensity of the voltage value. Thus, the results can be identified in the display panel. The RX part is paired with an Android smartphone via Bluetooth or USB. The generation of the TX signals is ordered by an app on the smartphone. The TX part generates the modulated signal by controlling the laser diode power digitally (on and off). The digitized pattern determines the line information with the protocol. The RX part detects the modulated signals, which are demodulated by measuring the time of high and low power through the photo diode. Hence, the matched protocol can identify the line information.

**Figure 3:**
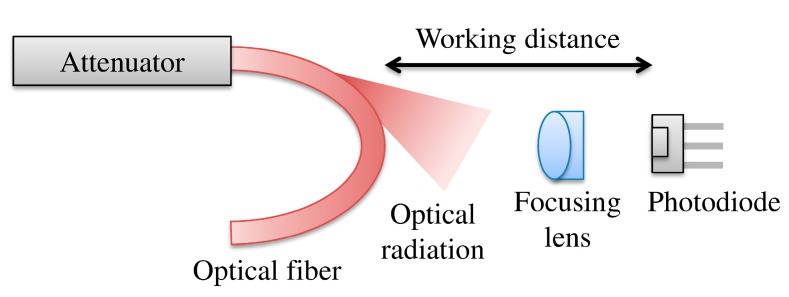
Performance improvement of handheld type information reader using a focusing lens.

The optical radiations of the fiber bends were simulated by the Synopsys, Inc. BeamPROP^®^ program. Two different simulations were conducted with respect to curvature of the fiber bending and wavelength. Then, an experiment was conducted to improve the sensitivity of the line information reader using focusing lenses, as shown in Figure 3. The modulated signals of the TX part are weakened as the distance increases from the TX part. Thus, the improvement of the RX part’s sensitivity is more useful at longer distances. The TX part was connected with an attenuator. The focusing lens was placed between the curve of the optical fiber and the photo diode of the RX part. The alignment was optimized by a three-axis stage in each part. First, the maximum working distance between the optical fiber and photo diode was measured by lenses of different focal lengths, including 2.00 mm, 3.10 mm, 4.51 mm, and 8.00 mm, with a fixed value of the TX part. Second, the RX part measured the optical radiation value by the attenuation of the TX part using a focusing lens of focal length 4.51 mm. In addition, the optical radiation was measured by the same attenuation of the TX part without a lens. The attenuation values were 4 dB, 5 dB, and 8 dB at 20 mm, 15 mm, and 20 mm of the maximum working distance, respectively. Then, the improved SNR value was analyzed by comparison of the measurements.

## 3. Results and Discussion

The RX part was paired with an Android smartphone. The order of the signal generation was transmitted to the TX part using the app. The TX part generated the modulated signals. Then, an artificial bending of the test fiber was made to detect the radiation field of the fiber using the RX part. A bending radius of 20.00 mm is critical to detect line information without causing communication errors. The maximum distance of the measurement is almost 25 km. The precision of the distance is less than 3 m. The sensitivity was measured as 15 dBm, and the RX part detected the wavelength of the line information from 1625 nm to 1670 nm, which is different from the wavelength of communication transmission (1270 nm to 1610 nm).

**Figure 4:**
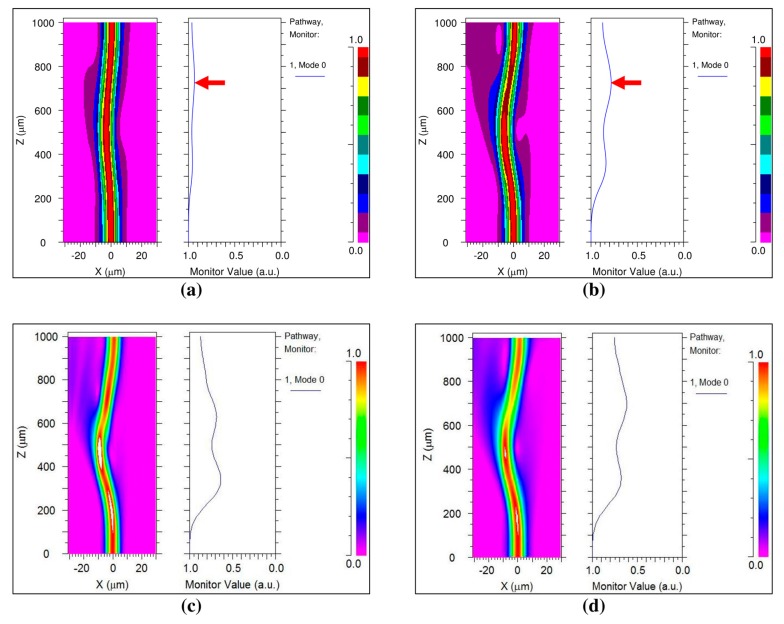
Optical radiation field of optical fiber at different wavelengths and radii of curvature: (**a**) Radius of curvature is 28 mm, the center of wavelength is 1625 nm; (**b**) Radius of curvature is 10 mm, the center of wavelength is 1625 nm; (**c**) Radius of curvature is 10 mm, center of wavelength is 1270 nm; (**d**) Radius of curvature is 10 mm, center of wavelength is 1625 nm.

The Synopsys, Inc. BeamPROP^®^ simulation of the optical radiation field is shown in Figure 4. Figure 4a,b are compared by the different radii of curvature in the same transmission wavelength. We used the discrete steps color bar to clearly see the leakage radiation of the propagation. Figure 4c,d are compared by the different transmission wavelengths in the same radius of curvature. We applied the continuous change of color bar to closely monitor the radiation and core signal of the transmitted signals. The magnitude represented the intensity of the transmission signals in the color bar. The X-axis is the horizontal length of the cross-sectional optical fiber, the Z-axis is the distance of the propagation, and the color bar means the intensity of the transmission signals. The monitor value is the optical signal value of the pathway in the core of the fiber. A smaller monitor value means that more radiation is leaked out from the bending of the optical fiber. For the first case, Figure 4a,b demonstrate that increased curvature of the fiber bending causes more leakage of the optical radiation of the bending under the same wavelength of 1625 nm. The left image of the fiber shows the optical radiation of the fiber, and the right graph shows the monitor value of the core in each figure. The applied radii of curvature are 28 mm and 10 mm in Figure 4a,b, respectively. The dark purple color indicates the optical radiation field from the bending of the fiber. The monitor value is the normalized value of the optical radiation in the core of the fiber. The minimum monitor value of the optical fiber (the point indicated by the red-colored arrow) is 0.95 and 0.8 in Figure 4b, respectively. The maximum loss of the transmission signal is 0.05 and 0.2 in Figure 4a,b, respectively. Thus, the radiation field of Figure 4b is four times larger than that of Figure 4a.

For the second case, Figure 4c,d identified that a longer transmission wavelength results in more leakage of the radiation field of the bending fiber than a short transmission wavelength under the same bending curvature. The radius of curvature was fixed at 10 mm in each figure. The radiation field of the fiber was simulated for transmission wavelengths of 1270 nm and 1625 nm in Figure 4c,d, respectively. The value of the radiation field from the bending of the fiber is 0.3 and 0.4 at 675 µm of the Z-axis in Figure 4c,d respectively. Therefore, more curvature of the bending and longer wavelength of the transmission signal cause more radiation field of the fiber, which was demonstrated in the simulations.

**Figure 5:**
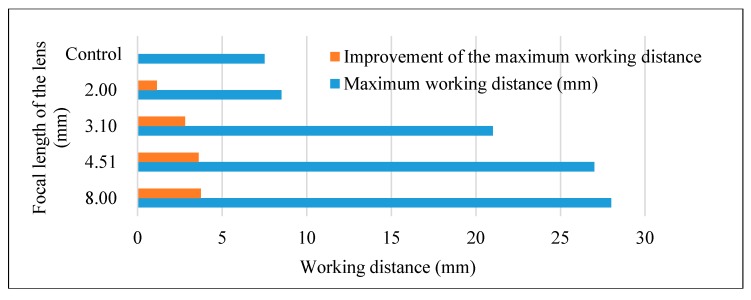
Working distance by employing different focusing lenses.

The experimental results are shown by using lenses of different focal lengths, as shown in Table 1 and Figure 5. The maximum working distance was 7.50 mm without lens and 8.50 mm, 21.00 mm, 27.00 mm, and 28.00 mm with lenses of focal length 2.00 mm, 3.10 mm, 4.51 mm, and 8.00 mm, respectively. The improvement of the maximum working distance (ratio of maximum working distance with lens and without lens) was 113%, 280%, 360%, and 373%, respectively. The lens of focal length 4.51 mm was chosen to apply the improvement sensitivity of the RX part, because that lens was considered as the most efficient by the size of the lens and the improvement rate.

The improvement in SNR is shown without lens and with the lens of focal length 4.51 mm at each of the different distances between fiber and photo diode in Table 2. The attenuation values without lens were 4 dB, 5 dB, and 8 dB when the distance between fiber and photodiode was 20 mm, 15 mm, and 10 mm, respectively. Similarly, the attenuation values with the lens of focal length of 4.51 mm were 10 dB, 11 dB, and 13 dB, respectively. The improved SNR values—which were calculated from the difference between columns (b) and (a) in Table 2—can be emphasized as 6 dB, 6 dB, and 5 dB. Thus, the sensitivity of the RX part was improved almost 6 dB by employing a focusing lens with a focal length of 4.51 mm.

## 4. Conclusions

We developed an optical line monitoring system by implementing a line information reader and line information generator for optical fiber communication networks. The proposed setup is applicable to examining and monitoring defaulted optical lines in optical networks. The line information generator is installed with bureau. Then, the handheld line information reader is applied to check optical line in OFD. The line information reader has 15 dBm sensitivity and a wavelength detection range from 1200 nm to 1700 nm. The line information generator transmits the modulated signals of the transmission wave from 1625 nm to 1670 nm. The radius of the bending curvature is 20 mm to detect the radiation field of the line information while not causing communication errors. The maximum working distance of the measurement is almost 25 km from the line information generator. The simulation of the Synopsys, Inc. BeamPROP^®^ program demonstrated that the smaller the radius of curvature and the longer the wavelength of the transmission optical signal, the more the optical radiation field leakage of the fiber bending. The sensitivity was improved by a factor of 3.6, and the SNR was enhanced by 6 dB by employing a focusing lens of 4.51 mm focal length. The line information generator transmits the modulated signals, and the line information reader separates the line conditions and information by demodulating the modulated signals. In conclusion, the developed optical monitoring system can be applied as a more efficient optical line information management solution in optical communication networks.

## Figures and Tables

**Table 1:** Working distance by employing different focusing lenses.

Focal Length of the Lens (mm)	Maximum Working Distance (mm)	Improvement of Maximum Working Distance (%)
-	7.50	-
2.00	8.50	113
3.10	21.00	280
4.51	27.00	360
8.00	28.00	373

**Table 2:** Improvement of the signal-to-noise ratio (SNR) by the different attenuations using the lens of focal length 4.51 mm.

Distance between Fiber and Photo Diode (mm)	Attenuation Value without Lens (dB) (a)	Attenuation Value with Lens of Focal Length 4.51 mm (dB) (b)	Improved SNR Value (dB) ((b)−(a))
20	4	10	6
15	5	11	6
10	8	13	5
